# Implementation of enhanced cognitive behaviour therapy (CBT-E) for adults with anorexia nervosa in an outpatient eating-disorder unit at a public hospital

**DOI:** 10.1186/s40337-018-0198-y

**Published:** 2018-05-29

**Authors:** Stein Frostad, Yngvild S. Danielsen, Guro Å. Rekkedal, Charlotte Jevne, Riccardo Dalle Grave, Øyvind Rø, Ute Kessler

**Affiliations:** 1 Department of Eating Disorders, Psychiatric Clinic, Haukeland University Hospital, Bergen, Norway; 2 Department of Clinical Psychology, University of Bergen, Bergen, Norway; 3 Department of Eating and Weight Disorders, Villa Garda Hospital, Garda, VR Italy; 4 Regional Department for Eating Disorders, Division of Mental Health and Addiction, Oslo University Hospital, Oslo, Norway; 5 Institute of Clinical Medicine, University of Oslo, Oslo, Norway; 6 Department of Clinical Psychiatry, University of Bergen, Bergen, Norway; 7 Psychiatric Department, Haukeland University Hospital, Bergen, Norway

**Keywords:** Anorexia nervosa, Adults, Cognitive behaviour therapy, Body mass index

## Abstract

**Background:**

Anorexia nervosa (AN) in adults is difficult to treat, and no current treatment is supported by robust evidence. A few studies, most of which were performed by highly specialized research units, have indicated that enhanced cognitive behaviour therapy (CBT-E) for eating disorders can be effective. However, the dropout rate is high and the evidence from non-research clinical units is sparse.

**Methods:**

This quality assessment project implemented CBT-E in an outpatient setting at a public hospital. Forty-four patients with AN started therapy. Each patient received at least 40 sessions of CBT-E over a 12-month period. Their body mass index (BMI) was recorded at baseline and after 3, 6 and 12 months. Reasons for not starting therapy or for leaving therapy prematurely were recorded.

**Results:**

Half (*n* = 22) of the 44 patients who started outpatient CBT-E did not complete the treatment. In the remaining sample there was a large (and statistically significant) weight gain after 12 months. The percentage of patients achieving the target BMI of > 18.5 kg/m^2^ was 36.4, 50.0 and 77.3% after 3, 6 and 12 months, respectively.

**Conclusions:**

This quality assessment project shows that it is possible to establish effective CBT-E in an outpatient eating-disorder unit at a public hospital. Although half of the patients did not complete CBT-E, the remaining patients achieved a significant increase in BMI at 1 year after the start of therapy.

## Plain English summary

Anorexia nervosa (AN) in adults is difficult to treat. Enhanced cognitive behaviour therapy (CBT-E) for eating disorders has shown promising effects in some studies. This outpatient method was implemented at a public hospital in Bergen, Western Norway. Half of the 44 patients who started CBT-E did not complete the treatment, but CBT-E was associated with significant and relatively large increases in body mass index in the remaining patients. This quality assessment project shows that CBT-E for AN can be implemented successfully in an outpatient setting at a public hospital.

## Introduction

Anorexia nervosa (AN) is a serious mental disorder with negative effects on physical, psychological and social functioning. The disorder is associated with high risks of severe medical complications and mortality [1]. While there has been some progress in treatments for children and adolescents with AN [2, 3], AN in adults still has a relatively poor prognosis [4, 5] and has been described as “one of the most difficult psychiatric disorders to treat” [6]. However, in recent years specific psychological interventions have shown promising results in some cohorts and randomized controlled trials.

Fairburn and colleagues studied 99 adults with AN from the UK and Italy who were treated with enhanced cognitive behaviour therapy (CBT-E) for eating disorders [7]. Their outpatient intervention was completed by 64% of the patients, who exhibited substantial improvements in weight and eating-disorder psychopathology. A variant of CBT-E has been compared with focal psychodynamic therapy and “optimized treatment as usual” in a multicentre randomized control trial involving 242 adults with AN [8]. All three treatments produced statistically significant improvements in mean body mass index (BMI), with no differences among them. In the Strong Without Anorexia Nervosa (SWAN) study, 120 patients with AN were randomized to 3 psychological treatments for AN: Specialist Supportive Clinical Management (SSCM), Maudsley Model Anorexia Nervosa Treatment for Adults (MANTRA), and CBT-E [9]. The treatments were completed by 60% of patients who showed equivalent effects on psychopathology and impairment. However, CBT-E was superior in helping patients to achieve a physically healthy weight, which is regarded as a fundamental requirement for recovery. These studies led to the recently published NICE (National Institute for Health and Care Excellence) guideline for eating disorders to recommend eating-disorder-focused cognitive behavioural therapy, MANTRA or SSCM for adults with AN [10].

Evidence-based psychological treatments are rarely implemented in clinical services for eating disorders in spite of these recommendations [11]. Moreover, when they are implemented, therapists usually fail to adhere to the manual or may even adopt an eclectic approach [12]. Problems with adherence to the manual are common in a real-world setting than in randomized control trials, which could reduce the therapeutic effects [11, 13].

Studies of the implementation of CBT-E as standard treatment for AN in non-research clinical settings are therefore needed to evaluate the utility of the treatment in normal clinical practice. The primary aim of this quality assessment project was to measure the pre-post changes in BMI in a sample of consecutive adult patients receiving outpatient CBT-E for AN.

## Methods

### Setting and design

This quality-improvement project was performed at the Department of Eating Disorders (DED) of the Psychiatric Clinic at Haukeland University Hospital, Bergen, Western Norway. The DED is a specialist eating-disorder unit that forms part of the public health-care system in Norway. The DED consists of a small inpatient unit and an outpatient unit. Referrals are accepted from specialist health-care institutions, but general practitioners in primary health care can also refer a patient suffering from AN directly to the unit if the severity of the eating disorder makes successful treatment in ordinary psychiatric care unlikely. The referrals are evaluated by all members of the treatment team in a weekly meeting. The criteria for acceptance to treatment at the DED are the presence of an eating disorder of clinical severity, prior unsuccessful treatment attempt in other specialist health-care institution or a severity that makes successful treatment in ordinary psychiatric care unlikely. All patients aged ≥16 years who fulfil the referral criteria as specified by guidelines from the Norwegian Health Authorities have the right to publicly funded treatment (all annual costs above 2500 NOK [250 euros] are covered).

The treatment was chosen based on the symptom severity and patients’ age. CBT-E [14] was the standard treatment for all patients > 18 years who did not require inpatient treatment (supportive weight normalization or intensive CBT-E [15]). Patients younger than 18 years were offered family-based treatment for AN (FBT) if they fulfilled the inclusion criteria for this treatment [16]. CBT-E [14] was offered as a standard psychotherapy intervention for adolescents who were unable to benefit from FBT.

Patients with severe psychiatric co-morbidity (e.g. substance misuse or active psychosis) that precludes them receiving focused eating-disorder treatment were referred to receive another treatment before the eating disorder is addressed at the DED. If the patient participated actively in outpatient CBT-E but was unable to gain weight, the patient was offered inpatient intensive CBT-E at the DED, as described elsewhere [15]. Patients who were unable to benefit from CBT-E and were developing a life-threatening condition could enter inpatient supportive weight-normalization treatment. In both such cases the outpatient CBT-E was regarded as not completed.

This quality assessment project performed a longitudinal evaluation of the implementation of outpatient CBT-E for AN in during 2013 and 2014. The patients did not receive any other eating-disorder psychotherapy while they were receiving CBT-E.

### Therapist training

The treatment team consisted of six clinical psychologists, one physician, one physiotherapist and one psychiatric nurse. All of the team members were trained CBT-E therapists who had attended a 2-day CBT-E workshop taken by the treatment developer Christopher Fairburn, followed by regular supervision by an experienced CBT-E psychotherapist. The team members also received weekly individual supervision from an experienced CBT-E therapist on-site during their first year at the DED. The implementation of CBT-E in individual patients is discussed in weekly 2-h team meetings. One of the main topics at these meetings is ensuring that all of the therapists adhere to the manual.

### The intervention

CBT-E is an individualized and flexible treatment specifically designed to address the eating-disorder psychopathology in the patient. The psychotherapy intervention has the following three main goals: (i) to remove the eating-disorder psychopathology (i.e. disturbed way of eating and low weight [if present]; extreme weight-control behaviours; and concerns about eating, shape and weight), (ii) to correct the mechanisms that have been maintaining the psychopathology specified in the patient’s formulation, and (iii) to ensure that the changes are long-lasting, by helping patients respond promptly to any setbacks [14]. The treatment is described in detail in the complete treatment guide [14].

The outpatient CBT-E for underweight patients is delivered individually by the same trained therapist over about 40 sessions, and it is organized into 3 main steps. In Step 1 the aim is to engage patients and help them arrive at the decision to regain weight as well as address the eating-disorder psychopathology. This step lasts up to 8 weeks and involves providing personalized education on the effects of being underweight, creating the formulation with emphasis on the role of low weight in maintaining the disorder and a focus on helping the patient to make the decision to change and regain weight. The patients participate in twice-weekly sessions until they consistently gain weight.

Step 2 focuses on achieving weight regain at the same time as addressing the key mechanisms that maintain the eating-disorder psychopathology. The goal is to help patients reach a body weight that can be maintained without dietary restriction and without symptoms of being underweight. This will allow a normal social life. For most patients these goals can be achieved with a BMI of 19.0–20.0 kg/m^2^. One session every 4 weeks is dedicated to reviewing the progress and the obstacles, and designing the subsequent 4 weeks of treatment.

Step 3 focuses on helping patients to maintain their weight. This step usually lasts for 8 weeks, with appointments towards the end of treatment occurring at intervals of 2–3 weeks. The aim is to ensure that progress is maintained and that the risk of relapse is minimized.

In addition to CBT-E, all underweight patients were advised to take standard dietary supplements: two omega-3 capsules, one 500-mg calcium tablets, and one multivitamin tablet daily. Patients with severe clinical depression were treated with fluoxetine or similar antidepressants.

### Assessment

#### Outcome measure

The primary outcome measure was the pre-post changes in BMI. Weight was measured using a balance beam scale while wearing normal clothing, and height was measured using a wall-mounted height board. Height and weight were measured by the individual therapists. If the BMI at the start of treatment was unknown, the BMI at referral was used as the baseline.

#### Demographic and illness characteristics

The following information was collected as part of the screening interview by the CBT-E therapists: age, gender, number of years with eating disorder before being referred to DED, other axis-I disorders and symptoms, previous treatments for eating disorders, living situation, marital status, occupation and whether the patient was on sick leave or receiving a disability pension.

#### Diagnostic evaluation

Eating disorders were diagnosed based on a clinical evaluation by an experienced psychologist or physician at the DED according to criteria in the Diagnostic and Statistical Manual of Mental Disorders, Fifth Edition (DSM-5) [17]. This study applied a BMI of < 18.5 kg/m^2^ as an inclusion criterion. The Mini International Neuropsychiatric Interview (MINI, version 6.0) [18] was used to screen for co-morbid psychiatric disorders at baseline. Suicidality was defined as reporting any suicidal thoughts or behaviours on the MINI.

#### Medical evaluation

The patients were assessed by a physician before they received health care at the DED. If the patient had severe AN, complications or co-morbid diseases, a senior medical specialist (S.F.) performed the medical assessment.

#### Reasons for not starting or not continuing treatment

If the patient decided to not start treatment, the therapist documented the background of this choice. Similarly, the reasons for ending prematurely were assessed in detail with patients during the sessions. Patients remaining in therapy for 12 months were regarded as completers.

### Statistics

Analyses were conducted using the IBM SPSS Statistics program (version 24). Paired-samples *t*-tests were conducted to compare the BMI between at the start of treatment (baseline) and after 12 months among the completers, as well as among all patients who started CBT-E (intention to treat – last observation carried forward). Cohen’s *d* effect sizes for within-sample changes in BMI from baseline to 12 months were calculated. Cohen’s *d* values of 0.2, 0.5 and 0.8 are considered to indicate small, moderate and large effects, respectively [19], while a value of 1.2 is considered to indicate a very large effect [20].

## Results

### Patient flow

During 2013 and 2014, 257 patients were referred to the DED, of which 108 referrals (42%) were not accepted. The main reason for not accepting a referral was that the patient had not received treatment at a general psychiatric outpatient unit (81% of all cases). Among the patients accepted for treatment, 78 (52%) were referred from primary health care and 71 (48%) were referred from specialized health care.

**Fig. 1:**
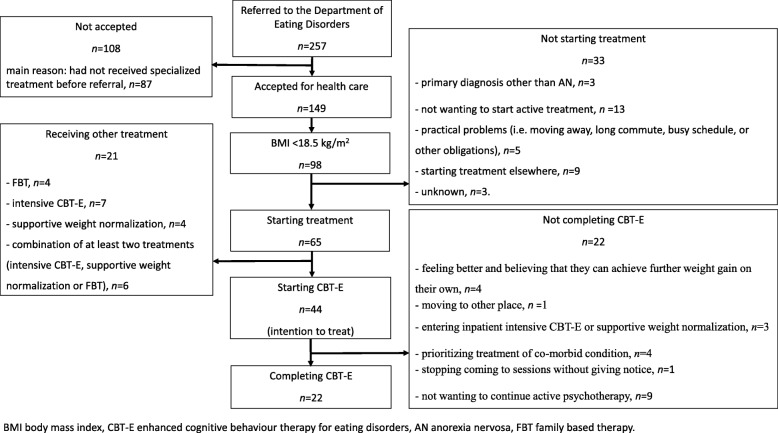
Flow chart over the patients referred to the Department of Eating Disorders during 2013 and 2014

A flowchart for the patients seeking treatment at the DED is shown in Fig. 1. Among the 149 patients 44 patients started CBT-E (the intention-to-treat sample); their sociodemographic background and illness characteristics are presented in Table 1. A considerable proportion of the patients struggled with suicidality thoughts or behaviours and depressive as well as anxiety disorders, as determined by the MINI (version 6.0) and clinical assessments.

**Table 1:** Characteristics of 44 consecutive patients starting CBT-E (enhanced cognitive behaviour therapy) for anorexia nervosa

Characteristic	All patients	Completers	Non-completers	*p*
	*n* = 44	*n* = 22	*n* = 22	
Age, years	23.3±6.9	25.6±8.4	21.1±4.2	0.030
Gender, male	1 (2.3%)	0	1 (4.5%)	
Number of years with ED	6.1 ± 6.0	8.0 ± 6.9	4.3 ± 4.3	n.s.
One or more other axis-I disorders^a^	25 (59.5%)	13 (61.9%)	12 (57.1%)	n.s.
Most-frequent other axis-I disorders and symptoms^a^				
Current major depressive episode	13 (31.0%)	8 (38.1%)	5 (23.8%)	n.s.
Recurrent major depressive episode	21 (50.0%)	13 (61.9%)	8 (38.1%)	n.s.
Suicidality (thoughts or behaviors)	25 (59.5%)	10 (47.6%)	15 (71.4%)	n.s.
Anxiety disorders	15 (35.7%)	8 (38.1%)	7 (33.3%)	n.s.
Previous treatment of ED^b^				
Previous inpatient treatment	13 (31.0%)	6 (27.3%)	7 (35.0%)	n.s.
Previous outpatient treatment in specialist health care	19 (45.2)	10 (45.5%)	9 (45.0%)	
Previous outpatient treatment in primary health care	6 (14.3%)	4 (18.2%)	2 (10.0%)	
No prior ED treatment	4 (9.5%)	2 (9.1%)	2 (10.0%)	
Living situation				
With one parent	6 (13.6%)	1 (4.5%)	5 (22.7%)	n.s.
With both parents	9 (20.5%)	3 (13.6%)	6 (27.3%)	
Alone	12 (27.3%)	7 (31.8%)	5 (22.7%)	
With partner	4 (9.1%)	1 (4.5%)	3 (13.6%)	
With partner and children	5 (11.4%)	3 (13.6%)	2 (9.1%)	
Without partner but with children	2 (4.5%)	2 (9.1%)	–	
Other	6 (13.6%)	5 (22.7%)	1 (4.5%)	
Marital status^c^				
Single	29 (67.4%)	15 (68.2%)	14 (66.7%)	n.s.
Girlfriend/Boyfriend	5 (11.6%)	3 (13.6%)	2 (9.5%)	
Partner/Co-habitant	5 (11.6%)	3 (13.6%)	2 (9.5%)	
Married	4 (9.3%)	1 (4.5%)	3 (14.3%)	
Occupation				
Full-time work	2 (4.5%)	1 (4.5%)	1 (4.5%)	n.s.
Part-time work	6 (13.6%)	3 (13.6%)	3 (13.6%)	
Labour-market measures	5 (11.4%)	2 (9.1%)	3 (13.6%)	
School student/apprentice	10 (22.7%)	3 (13.6%)	7 (31.8%)	
College/University student	17 (38.6%)	11 (50%)	6 (27.3%)	
On sick leave	3 (6.8%)	2 (9.1%)	1 (4.5%)	
Disability pension	0	0	0	
Other	1 (2.3%)	0	1(4.5%)	
Baseline body mass index	16.3±1.6	16.4±1.9	16.2±1.3	n.s.

Data are mean±SD or *n* (%) values

*ED* eating disorder, *n.s.* not significant

^a^*n* = 42 (21 completers, 21 non-completers)

^b^*n* = 42 (22 completers, 20 non-completers)

^c^*n* = 43 (22 completers, 21 non-completers)

### Intention-to-treat findings

The BMI increased from 16.3±1.6 kg/m^2^ (mean±SD) at baseline to 18.3±2.2 kg/m^2^ (last observation carried forward) among all the patients who started treatment (*p* < 0.001). Cohen’s *d* for this change was 1.0 and thus it was classified as a large effect. The percentage of patients presenting with BMI ≥ 18.5 kg/m^2^ at the last observation was 54.5% (24 of 44 patients).

### Proportion of patients who completed CBT-E

Half of the patients (*n* = 22) completed the treatment, while 22 patients (50%) ended the treatment prematurely for reasons listed in Fig. 1. Completers were significantly older than non-completers, while their BMI, number of years with eating disorder, rate of psychiatric co-morbidity, number of previous eating-disorder treatment attempts and living situation were all similar (as listed in Table 1).

### Outcomes among completers

**Fig. 2:**
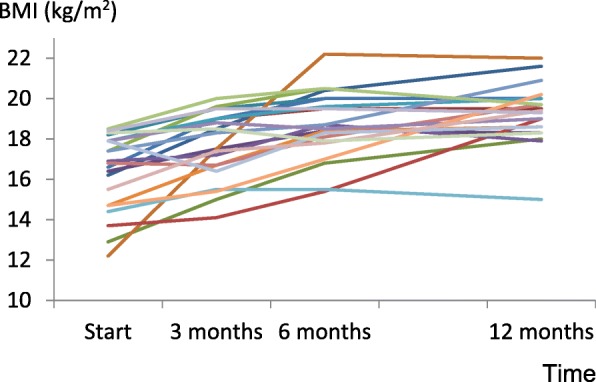
BMI over the course of treatment for 22 patients who completed CBT-E

The BMI over the course of treatment for the 22 completers is shown in Fig. 2: it was 16.4±1.9, 17.7±1.7, 18.7±1.6 and 19.3±1.4 kg/m^2^ at baseline and after 3, 6 and 12 months, respectively. There was a significant weight gain after 12 months (BMI difference of 2.9±2.3 kg/m^2^, range 0.0–9.8 kg/m^2^, *p* < 0.001). The effect size for this change was very large (Cohen’s *d =* 1.7) and the percentage of patients achieving the target BMI of ≥18.5 kg/m^2^ was 36.4, 50.0 and 77.3% after 3, 6 and 12 months, respectively.

### Outcomes among non-completers

The BMI over the course of treatment for the 22 non-completers was 16.2±1.3, 17.2±1.9, 18.0±1.5 and 18.3±2.2 kg/m^2^ at baseline and after 3, 6 and 12 months, respectively. There were missing data from several patients at 3, 6 and 12 months, and the weight gain and effect size of this change was therefore not computed. The percentage of patients presenting with BMI ≥ 18.5 kg/m^2^ at the last observation was 31.8% (*n* = 7).

### Outcome among patients with severe AN

Seventeen of the 44 patients who started CBT-E presented with severe AN (BMI < 16 kg/m^2^ according to DSM-5), of which 7 were completers. Five of these seven patients had a BMI of ≥18.5 kg/m^2^ at 12 months after starting CBT-E: the BMI in this group of patients was 14.0±1.1 kg/m^2^ at baseline and 18.9±2.1 kg/m^2^ after 12 months.

## Discussion

This quality assessment study aimed to describe the pre- post changes in BMI in a sample of consecutive patients treated with CBT-E for AN at a specialized outpatient eating-disorder unit at a public hospital. There were two main findings: (i) more than two-thirds of the patients who completed the treatment achieved a normal weight after 12 months, and (ii) half of the patients ended the treatment prematurely and did not recover to the same level as those who completed the treatment. In addition, there was a relatively large effect on BMI in this outpatient setting among a substantial subgroup of the patients with severe AN. These are typical patients who usually are referred to inpatient care or other intensive medical stabilization treatments, and not to outpatient psychological treatment. The implementation of CBT-E for AN allowed patients who previously would have been treated as inpatients to live their ordinary lives while they were receiving treatment.

CBT-E for AN was relatively easy to implement in our hospital outpatient unit. Moreover, the results of the present quality-assessment study are promising and are in line with those reported for clinical trials that have assessed the efficacy of CBT-E [9, 14].

The main problem to address in the future is to reduce the proportion of non-completers. Indeed, the percentage of non-completers was higher than both that for CBT-E in the study in UK and Italy (36.4% were non-completers) and in the CBT-E arm of the SWAN study (33.3% were non-completers), while it was identical to that reported for an Australian effectiveness study on CBT-E in patients with AN [21]. While 77% of the patients who completed the treatment achieved the target BMI, the high dropout-rate implies that this represents only 39% of all the patients starting CBT-E. However, it should be stated that the rate of non-mutual premature termination of treatment was only 34% in the current sample, which is similar to reported rates for research clinical trials; seven of our patients were referred by our team to intensive treatments or to address other co-morbid conditions. It is also possible that some elements of randomized clinical trials missing in our clinical settings—such as excluding patients with severe AN and actively recalling patients who missed some sessions—might explain the non-completer rate being higher for our treatment than for CBT-E research trials.

Several studies have indicated that it is difficult to identify reliable predictors of attrition [22, 23]. In our sample non-completers were younger than completers (25.6 versus 21.1 years *p* = 0.03), suggesting that CBT-E might be more suitable for older patients. However, there were adolescents in both groups, and CBT-E has shown to be a promising treatment also for adolescents with AN [3]. Although we have no data indicating how to reduce attrition when implementing CBT-E in a real world clinical setting, the clinical experience that we gained by this quality improvement project leads us to suggest the following strategies on how to reduce treatment attrition. First, more time and effort should be dedicated to prepare the patients for CBT-E, stressing the importance of giving treatment priority, playing an active role and completing the treatment. Second, a great store should be placed on establishing and maintaining therapeutic momentum, stressing the importance to avoid breaks in treatment. Third, since patients with AN come to treatment with varying degrees of reluctance and ambivalence engaging the patient should be the top priority for the entire course of the treatment. Further, factors related to the treatment process itself (such as therapeutic alliance and early patient engagement in the treatment) warrant attention, and should be investigated in future studies [24].

This study was subject to the following limitations: there were missing data, especially from patients dropping out from treatment; no systematic data were obtained on pre-post changes in eating-disorder symptomatology or on a possible diagnostic switch to bulimia nervosa; and we obtained no long-term data describing the clinical situations of the patients after the 12-month assessment. We also did not assess therapist competence and treatment fidelity. However, since treatment fidelity was regarded as crucial for treatment success and essential for the feasibility of setting up this treatment, during the weekly 2-h team meetings adherence to the manual was regularly addressed. However, the main strength of the study is that it demonstrated the possibility of effectively implementing an evidence-based outpatient treatment for AN in a real-world clinical setting.

## Conclusion

CBT-E can be implemented relatively easily in an outpatient setting at a public hospital. Patients who remain in therapy are likely to exhibit a substantial increase in BMI and thereby avoid costly and life-disruptive inpatient treatments. However, a large subgroup of patients does not complete the treatment, and the most challenging problem for future research to address is decreasing the non-completion rate.

## Abbreviations

AN: Anorexia nervosa
BMI: Body mass index
CBT-E: Enhanced cognitive behaviour therapy
DED: Department of Eating Disorders
DSM-5: Diagnostic and Statistical Manual of Mental Disorders, Fifth Edition
FBT: Family based treatment
IBM SPSS: International Business Machines Statistical Package for the Social Sciences
MANTRA: Maudsley Model Anorexia Nervosa Treatment for Adults
MINI: Mini International Neuropsychiatric Interview
NICE: National Institute for Health and Care Excellence
NOK: Norwegian Krone
REK: Regional Ethical Committee
SSCM: Specialist Supportive Clinical Management
SWAN: Strong Without Anorexia Nervosa Study
UK: United Kingdom
